# A Clinical and Pathological Variant of Acute Transplant Glomerulopathy

**DOI:** 10.1155/2014/961987

**Published:** 2014-09-11

**Authors:** Miklos Z. Molnar, G. V. Ramesh Prasad, Darren A. Yuen, Serge Jothy, Jeffrey S. Zaltzman

**Affiliations:** ^1^Division of Nephrology, Department of Medicine, University of Tennessee Health Science Center, Memphis, TN 38152, USA; ^2^Renal Transplant Program, St. Michael's Hospital, University of Toronto, Toronto, ON, Canada M5B 1W8; ^3^Keenan Research Centre of the Li Ka Shing Knowledge Institute, St. Michael's Hospital, Toronto, ON, Canada M5B 1W8; ^4^Department of Laboratory Medicine, St. Michael's Hospital and Department of Laboratory Medicine and Pathobiology, University of Toronto, Toronto, ON, Canada M5S 1A1; ^5^Division of Nephrology, St. Michael's Hospital, 61 Queen Street East, Room No. 9-118, Toronto, ON, Canada M5C 2T2

## Abstract

Acute transplant glomerulopathy transplant glomerulopathy (TG) is a common cause of late renal allograft loss. We describe a unique case of a renal transplant recipient who developed rapid-onset nephrotic-range proteinuria and acute kidney injury secondary to C4d negative acute TG. Two courses of intravenous Rituximab resulted in significant improvement in proteinuria and allograft function. In the setting of acute nephrotic-range proteinuria postrenal allograft, both renal biopsy with electron microscopy and screening for de novo donor-specific antibody should be performed to distinguish atypical presentations of TG from other diagnoses.

## 1. Background

The prevalence of late renal allograft loss remains high, with the well-known recognition that donor-specific alloantibody (DSA) plays a role in this problem. One important phenotype is transplant glomerulopathy (TG), also called transplant glomerulitis, which has been recognized in 1973 [1].

The main mechanism of TG is alloantibody-mediated injury of the endothelial cells [2]. Other etiologies of transplant-associated glomerulitis include hepatitis C virus infection, autoimmunity (lupus), indolent thrombotic microangiopathy [3], and T cell-mediated rejection [4]. Most often, TG is recognized in its chronic form, although acute clinical presentations requiring a biopsy are known to occur. Therefore, what is known from the pathology of TG is derived from biopsies taken during the advanced stage of glomerulopathy. Conversely, much less is known about the pathogenesis and pathology of the early phase of TG.

The diagnosis of TG may be suspected clinically; however, there is no single pathognomonic characteristic of TG. Worsening graft function, proteinuria, and hypertension are the main findings. Significant postrenal transplant proteinuria with concurrent allograft dysfunction can be secondary to recurrent glomerulopathy (such as IgA nephropathy, membranous nephropathy, or focal segmental glomerulosclerosis (FSGS)) and de novo glomerulonephritis (such as membranous nephropathy or FSGS or drug side effects) [5]. Confirmation of suspected TG requires a biopsy of the allograft. However, light microscopy and C4d immunostaining may not be sufficient to establish a diagnosis. A recent case series showed that only 55% of biopsy specimens with confirmed TG had C4d positivity in the peritubular capillaries (PTC), while 76% of the patients had anti-HLA antibody, of which 54% were donor specific [6].

We present a case of abrupt development of substantial nephrotic-range proteinuria and deterioration of graft function secondary to C4d negative acute TG 16 months after transplantation.

## 2. Case Report

A 46-year-old male with end stage renal disease (ESRD) secondary to IgA nephropathy received a neurological determination of death (NDD) deceased donor kidney transplant in April 2012.

The donor was Hepatitis B core antibody positive but negative for Hepatitis B surface antigen and Hepatitis C. At the time of transplantation, the recipient was of low immunological risk, with calculated panel reactive antibody (cPRA) of 0% and no DSA. Immunosuppressive therapy included 20 mg basiliximab preoperatively and on postoperative day 4, with intravenous Solu-Medrol 1 g/kg preoperatively and 1 g/kg every 12 hours for 48 hours, followed by an oral prednisone taper to 5 mg daily by 60 days following transplant. His initial immunosuppressive regimen also included once daily Tacrolimus with Tacrolimus levels between 4 and 7 ng/mL and mycophenolate-mofetil (MMF) 1 gram twice a day.

His postoperative course was uncomplicated and he was discharged from hospital with a baseline creatinine of 1.25 mg/dL. In January 2013, 9 months after transplant, he was found to be positive for BK viremia by routine surveillance PCR. MMF was reduced to 500 mg daily with clearance of the virus by September 2013. Prior to that time, monthly urine albumin/creatinine ratios were always normal and his blood pressure was managed with a single antihypertensive agent.

**Figure 1:**
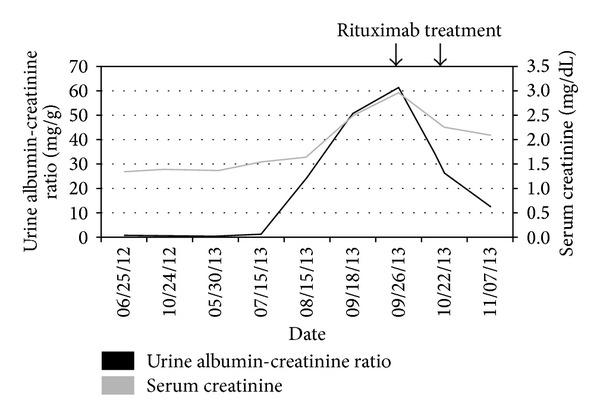
Time course of proteinuria and serum creatinine.

At 16 months after transplant, September 2013, his stable hypertension became difficult to manage. Shortly thereafter, he developed new onset, nephrotic-range proteinuria at 6.2 g/24 hours, along with worsening blood pressure and deteriorating allograft function with a creatinine of 2.95 mg/dL. The laboratory parameters, including serum creatinine and urine albumin-creatinine ratio, are depicted in Figure 1.

**Figure 2:**
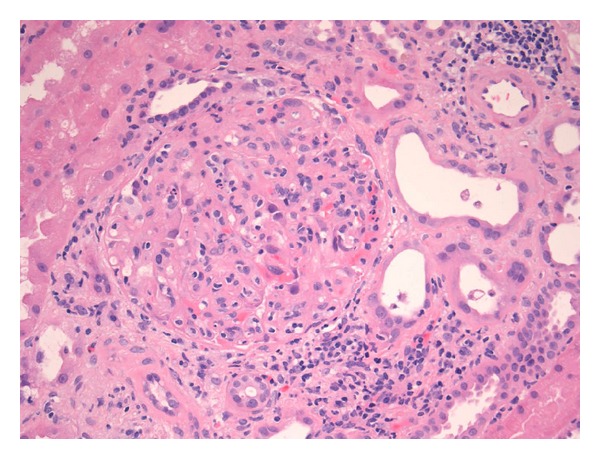
The glomerulus is hypercellular, with an increased number of polymorphonuclear and mononuclear inflammatory cells (hematoxylin and eosin stain).

**Figure 3:**
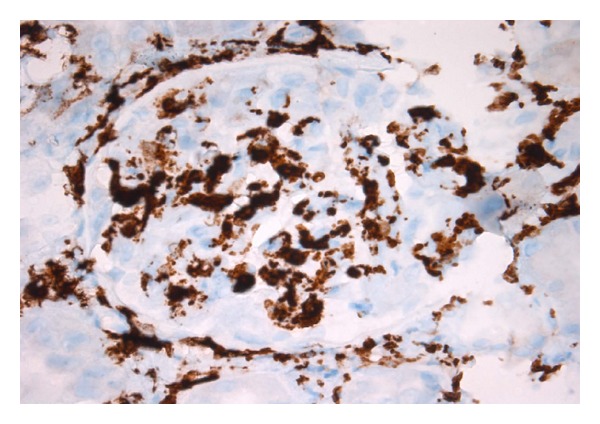
A large number of glomerular cells stain positively for the CD163 monocyte marker (immunohistochemistry).

**Figure 4:**
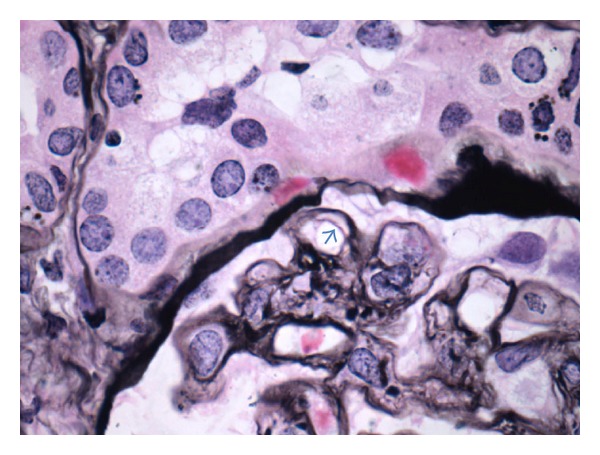
Duplication of the GBM is present in some glomerular capillary loops (green arrow) (Jones methenamine silver stain).

**Figure 5:**
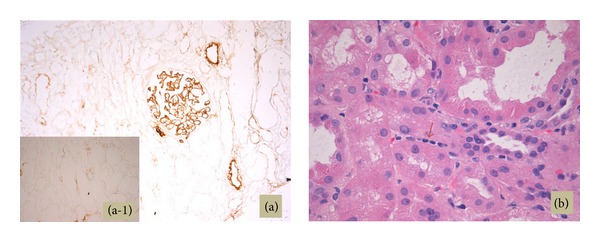
C4d immunostaining shows no staining of interstitial capillaries. There is positive staining of glomeruli and arteries endothelial lining, which is a normal internal control finding on cryostat sections immunostained with the C4d antibody (Quidel, San Diego, CA) ((a) including high magnification inset (a-1)) (immunohistochemistry). Only rare capillaritis was observed ((b) red arrow) (hematoxylin and eosin stain).

A renal allograft biopsy was performed in October 2013. On light microscopy, there were 52 glomeruli in the specimen. No glomeruli were globally sclerosed. Many glomeruli were hypercellular mostly because of the presence of leucocytes in some glomerular capillary loops (Figure 2). The endothelial cells were swollen. There were a high number of monocytes in endocapillary locations of the glomeruli, as shown by CD163 immunohistochemical staining (Figure 3). The peripheral capillary wall was noted to be thickened in some glomeruli. Using the hematoxylin and eosin stain, no conspicuous duplication of the glomerular capillary wall was noticed. However, the Jones methenamine silver stain showed occasional capillary loops with GBM duplication (Figure 4). There was significant inflammation of the interstitium with lymphocytes and polymorphonuclear leucocytes. Peritubular inflammation was present but there was no tubulitis. Arteries were present in the biopsy and one of them had mild endarteritis. There was no evidence of thrombotic microangiopathy. Minimal peritubular capillaritis (Figure 5(b)) and mild focal interstitial fibrosis were present. By Banff criteria, the light microscopy findings were classified as g1, i2, t0, and v1.

**Figure 6:**
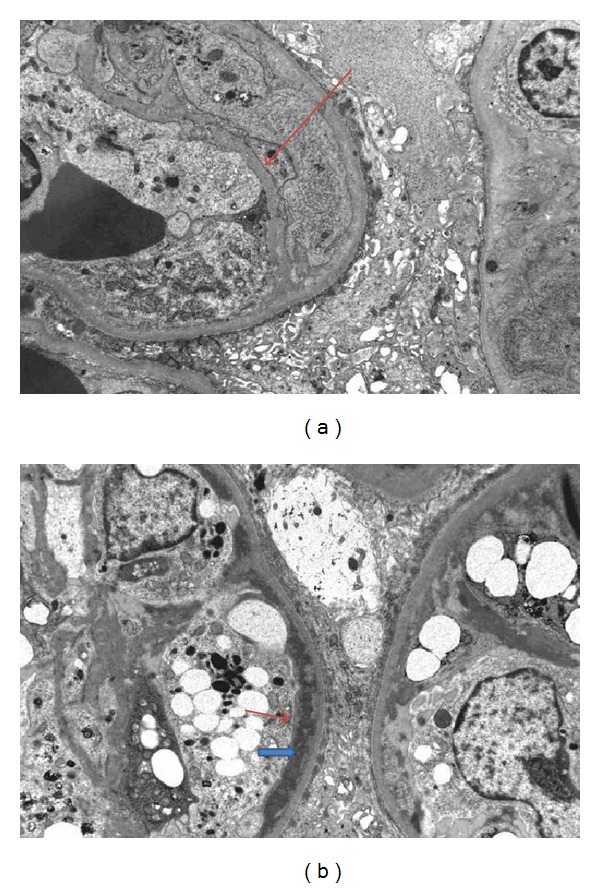
Electron micrograph of a glomerulus showing duplication of the GBM ((a) red arrow). Small subendothelial electron dense deposits ((b) blue arrow), underlaid by a thin layer of duplicated GBM ((b) red arrow), are also present (electron microscopy).

There was no C4d immunostaining in the interstitial capillaries (Figure 5(a)). Immunofluorescence showed 1+ peripheral finely granular IgM and IgA and lambda staining in the glomeruli. No significant immunostaining for C3 was noticed. There was no mesangial IgA staining. Trace peripheral staining for fibrinogen was present. By electron microscopy (EM), occasional glomerular capillary loops had duplication of the GBM (Figure 6). There were also occasional small electron-dense deposits in subendothelial location (Figure 6). There were no hump-like subepithelial deposits or mesangial deposits. The mesangial matrix was expanded. A prominent feature was expansion of the subendothelial space of the peripheral capillary loops with marked glomerular endothelial cell swelling, equivalent to endotheliosis, occluding a significant part of the glomerular capillary lumens. The podocytes had fused foot processes over several segments of the peripheral capillary loops.

In summary, the microscopic and electron microscopic changes were suggestive of C4d negative TG. After the biopsy results were available, the serologic DSA came back as positive for de novo class II DSA against DQA3.

Following diagnosis, the patient received 2 courses of intravenous Rituximab 375 mg/1.73 m^2^. After the first two doses of Rituximab, the proteinuria decreased to ~1 g/day and his serum creatinine improved from 2.95 to 2.09 mg/dL (Figure 1). The hypertension was treated with Diltiazem which resulted in normalization of the blood pressure.

## 3. Discussion

Transplant glomerulopathy is one of the most common causes of renal allograft loss. Rare before 6 months, the incidence increases from 4.0% at 1 year to 20.2% at 5 years [7]. Here, we describe a case of sudden onset nephrotic-range proteinuria (>6 g/24 hours) with hypertension and rapid deterioration of graft function secondary to C4d negative TG only 16 months after transplantation. The diagnosis of acute TG is supported by the hypercellular glomerulopathy with a high number of monocytes in the glomeruli and early duplication of the GBM and clinically by the presence of DSA.

The negative staining for C4d in the interstitial capillaries does not rule out a diagnosis of TG as this negative finding was also reported in a high proportion of patients with acute TG (4–6, 15).

Transplant glomerulopathy is a very strong predictor of poor long-term outcome in kidney transplant recipients. Data from a large series of cross-match negative kidney recipients showed 62% graft survival for those with TG as opposed to 95% graft survival for those without TG at 5 years following transplantation [8]. Graft survival rate is further affected if TG is associated with C4d deposition in the PTC [9]. De novo DQ DSA is also an established prognostic factor of poor graft outcome, which may be ameliorated with DQ matching.

Our case is unique in several ways. Firstly, the amount of proteinuria was more than 6 g/24 hours. Secondly, the nephrotic-range proteinuria developed acutely as the patient had had normal urine albumin/creatinine ratios previously by monthly sampling. While >1 g/24 hours of proteinuria can be seen in patients with TG, in most studies of TG, nephrotic- range proteinuria developed in 29% of patients [10]. More than 5 g/24 hours of proteinuria is more commonly observed in recurrent or de novo glomerulonephritis [5]. To the best of our knowledge, no previous case of such marked proteinuria with TG has been described [11]. The acute TG developed after a delay of 16 months following kidney transplantation. Although early, in a retrospective study of 55 patients with the usual type of TG, the mean time from transplantation to diagnosis of TG was 21 months (range: 4–61 months) [12, 13]. Finally, although small and scarce, the presence of subendothelial electron-dense deposits is another finding in this case. Considering that no deposits were found in mesangial location, this finding is unlikely related to recurrent IgA nephritis. Instead, it could represent an unusual early change in the pathogenesis and the subendothelial deposition could act as a trigger for the duplication of the GBM.

The diagnosis of chronic cases of TG often can be established by light microscopy findings of prominent duplication of the glomerular basement membrane (GBM) and mesangial matrix expansion with no or mild mesangial hypercellularity. The glomerular involvement can be focal or diffuse according to the severity of the disease [14]. In typical TG, there is a lack of significant IgG, IgA, and C1q staining in glomeruli by immunofluorescence. However, positive C4d immunostaining is commonly seen in chronic TG [15]. Patients with the chronic type of TG typically have capillary C4d deposition and detectable DSA [12, 13].

Early diagnosis of TG, however, requires electron microscopy [3]. Nair et al. described 3 cases of very early acute TG with endotheliosis as the predominant feature; however, unlike our case, the associated proteinuria in these 3 patients was considerably less [16]. However, early lesions of TG seen only by EM (as was the case here) may actually be seen as early as during the first few months after transplantation [17, 18].

The best treatment option for TG has not yet been established. The removal or suppression of antibody production is a treatment option for TG. Previous studies assessed the role of Rituximab, a chimeric monoclonal antibody against CD20 that depletes B cells. In a recent pediatric randomized-controlled trial, 45% of patients with TG responded to a combination of IVIG and Rituximab treatment [19]. Interestingly, our patient responded well to only 2 courses of Rituximab treatment. However, we do not have data about DSA level after our treatment.

In summary, we have presented a case of acute transplant glomerulopathy characterized by rapid development of nephrotic-range proteinuria, hypertension, and allograft function deterioration secondary to C4d negative TG. The patient responded well to therapy with Rituximab.
